# Unraveled: The role of Arabidopsis GTE4-EML in transcription via two distinct histone modifications

**DOI:** 10.1093/plcell/koaf009

**Published:** 2025-01-06

**Authors:** Pei Qin Ng

**Affiliations:** Assistant Features Editor, The Plant Cell, American Society of Plant Biologists; Department of Plant Sciences, University of Cambridge, Cambridge CB2 3EA, UK

The initiation of transcription depends on the activity of chromatin remodeling complexes that loosen or unravel the dense chromatin structure at the transcription start site, allowing the transcriptional machinery to bind and carry out transcription. GLOBAL TRANSCRIPTION FACTOR GROUP E4 (GTE4) is a versatile chromatin remodeling factor in Arabidopsis (*A. thaliana*) that has been shown to regulate mitotic cell division (Airoldi et al. 2010) and influence plant immunity (Zhou et al. 2022). GTE4 consists of a bromodomain and extra terminal domains that recognize histone acetylation, a key chromatin mark that loosens chromatin structure (Airoldi et al. 2010). GTE4 function appears to overlap with that of other chromatin modulators, such as EMSY-LIKE PROTEINs (EMLs), which recognize histone H3K4 trimethylation and also play vital roles in seed development and plant immunity (Coursey et al. 2018; Milutinovic et al. 2019; Tsuchiya and Eulgem 2011). In plants, it is unclear how 2 of these distinct histone modifications—acetylation and trimethylation—crosstalk to co-regulate transcription initiation. **Qian and colleagues (Qian et al. 2024)** identify GTE4 as a key player in this crosstalk via direct interaction with EML1/2.

Qian et al. (2024) investigated the histone acetylation binding capability of Arabidopsis GTE4 and found strong binding affinity to H4 acetylation (H4K5/8/12/16ac) but weaker affinity to H3 acetylation lysines (H3K4/9/14/18ac) and monoacetylated histones. To identify the co-interactors of GTE4, the authors generated plants expressing FLAG epitope-tagged GTE4. They found that EML1/EML2 co-precipitated with GTE4 (and vice versa), confirming the GTE4-EML1/2 interaction. Yeast 2-hybrid experiments confirmed that EML1 and EML2 co-interact, suggesting the formation of GTE4-EML1/2 complex to facilitate active transcription.

Through yeast 2-hybrid screening using truncated GTE4 protein, they identified the domain responsible for the GTE4-EML1 interaction, which is predicted to form a conserved alpha-helix. Affinity purification—mass spectrometry confirmed that the deletion of this domain disrupts the interaction of both EML1 and EML2 with GTE4, which the authors further confirmed using co-immunopurification. Compellingly, the authors observed that the *eml1 eml2* double mutant displayed a similar mutant phenotype to the *gte4* mutant but no mutant phenotype in the *eml1* or *eml2* single mutants, further supporting a shared role of EML1 and -2 in the GTE4 interaction (Fig.). From multiple chromatin immunoprecipitation sequencing (ChIP-seq) experiments, the authors found that GTE4-EML occupies genes enriched with histone acetylation and H3k4me3.

**Figure.**
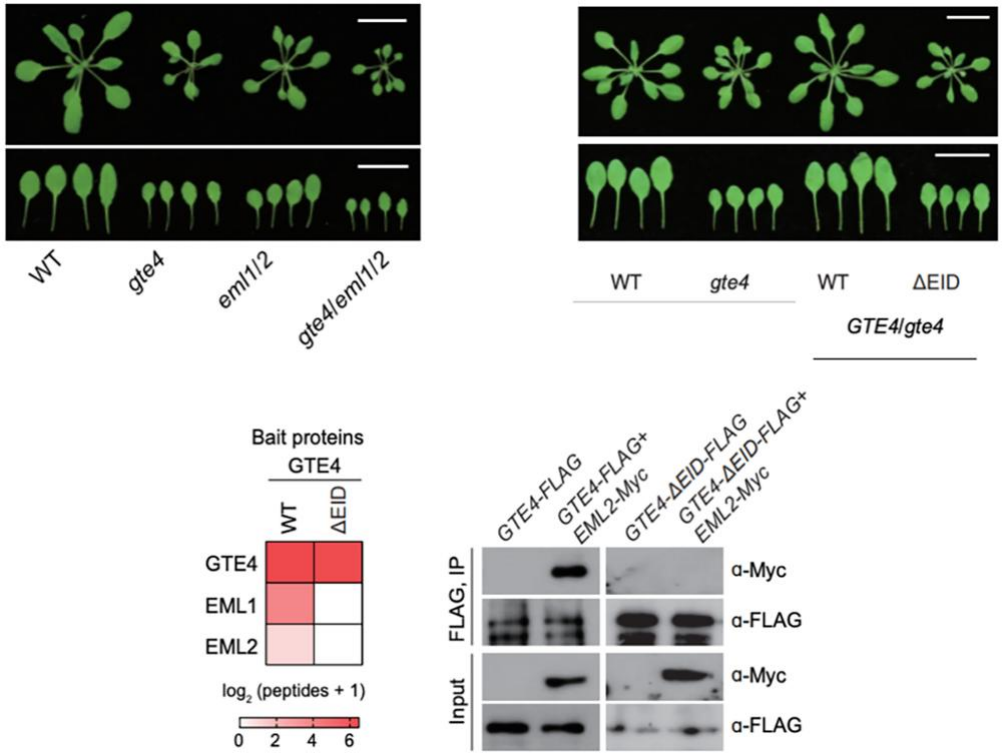
Interaction between GTE4 and EML1/EML2. (Top left) Phenotypes of *gte4, eml1/2* double mutant, *gte4/eml1/2* triple mutant; (top right) loss of functional domain leads to mutant phenotype similar in gte4; (bottom panel) loss of functional domain leads to loss of EML1 and EML2 affinity to GTE4. Figure adapted from Qian et al. (2024), Figs. 2 and 3.

In summary, Qian et al. (2024) work elucidated the formation of a protein complex, GTE4-EML, that facilitates the co-recognition of 2 different histone modifications. This work sheds light on the intricate transcription initiation processes in plants.

## Data Availability

No new data were generated or analysed in support of this article.

## Dive Curated Terms

The following phenotypic, genotypic, and functional terms are of significance to the work described in this paper:

EML2 Gramene: AT5G06780
EML2 Araport: AT5G06780

GTE4 Gramene: AT1G06230
GTE4 Araport: AT1G06230

EML1 Gramene: AT3G12140
EML1 Araport: AT3G12140
